# Sustainable transformation agenda for enhanced global food and nutrition security: a narrative review

**DOI:** 10.3389/fnut.2023.1226538

**Published:** 2023-08-02

**Authors:** Oluwatoyin Oluwole, Olubunmi Ibidapo, Temiloluwa Arowosola, Fatima Raji, Renata Puppin Zandonadi, Ibrahim Alasqah, Linda Heejung Lho, Heesup Han, António Raposo

**Affiliations:** ^1^Department of Food Technology, Federal Institute of Industrial Research, Oshodi, Lagos, Nigeria; ^2^University of Brasília, Faculty of Health Sciences, Nutrition Department, Campus Universitário Darcy Ribeiro, Brasília, Brazil; ^3^Department of Public Health, College of Public Health and Health Informatics, Qassim University, Al Bukairiyah, Saudi Arabia; ^4^Division of Tourism and Hotel Management, College of Business, Cheongju University, Cheongju-si, Chungcheongbuk-do, Republic of Korea; ^5^College of Hospitality and Tourism Management, Sejong University, Seoul, Republic of Korea; ^6^CBIOS (Research Center for Biosciences and Health Technologies), Universidade Lusófona de Humanidades e Tecnologias, Lisboa, Portugal

**Keywords:** food security, sustainability, nutrition, developing countries, global food

## Abstract

The world’s population is currently growing at an exponential rate, which is estimated to be over 8 billion inhabitants as reported by United Nations on November 15, 2022. According to FAO, 870 million people out of this population do not have enough food to eat, with the vast majority of hungry people (98%) living in developing countries, where almost 15% of the population is undernourished. Furthermore, the world’s population is estimated to come to 9 billion by 2050, which would incur severe food scarcity and would seriously hamper global food security. Food losses, especially post-harvest loss as a result of poor agricultural practices have also been reported to greatly impact the economy, the environment, and the livelihoods of low and medium countries and Sub-Saharan-African. Therefore, realistic options should be established for promoting sustainable agriculture systems, improving nutrition, and achieving food security to end hunger in our nations. This paper elucidates the drivers of food insecurity including food losses, escalating population growth, hunger, and food production, among others, and provided some transformation approaches such as value addition through appropriate and emerging food processing and preservation techniques, application of biotechnological options through genetically modified foods and functional foods consumption and integration of indigenous underutilized nutrient-dense food crops which could serve as all-inclusive and sustainable transformation options for enhanced food and nutrition security, especially in developing countries, which is where the hunger burden and the prevalence of malnutrition and non-communicable diseases are high.

## Introduction

The FAO (Food and Agriculture Organization) defines food security to be a condition when “all people at all times have physical and economic access to sufficient, safe, and nutritious food to meet their dietary needs and food preferences for an active and healthy life” (1). Food security addresses food availability, food utilization, food access and food stability, whereas nutrition security focuses on food utilization in the body, and on individual and household food consumption (2). The FAO (3) defines nutrition security as a situation that arises when secure access to a suitably nutritious diet is accompanied by a good sanitary environment, adequate health services and care that guarantees a healthy active life for all household members. The relationship between food safety and food/nutrition security must be strengthened to achieve enhanced global food-secured systems. Food safety entails good food handling procedures and practices to be applied while preparing food, and during the processing, storage, and distribution of products to prevent foodborne illness. Reports show that unsafe food contains parasites, viruses, harmful bacteria and/or chemical substances because over 200 diseases, globally which range from diarrhea to cancers, and good hygiene practices in the food and agricultural sectors would drastically help to reduce the emergence and spread of foodborne diseases (4). In other words, diet quality is an essential link between food and nutrition security. Poor diet quality and safety can result in distinct disease forms like malnutrition, which includes micronutrient deficiencies and undernutrition. It also contributes to overweight and obesity which, in turn, contributes to food insecurity.

The 2020 COVID-19 pandemic negatively affected the food and nutrition security of almost all the world’s nations globally. Access to food and increased food costs affected almost everyone, but more profoundly affected people from low−/middle-income countries (5). Recently, a review revealed that food accessibility was the most affected food security dimension, with evidence for food affordability being severely impacted by most families’ reduced purchasing power. Unfortunately, evidence that food availability was affected beyond certain initial disruptions is not available, which was due to “panic buying.” There is no compelling evidence of the effects of the COVID-19 pandemic on food safety and quality. Nevertheless, access or availability disruptions disturb the stability dimension of food security. Household food insecurity increased in Nigeria to approximately 77% of the post-COVID-19 level in 2020 compared to 51% in 2018, which was the pre-COVID-19 level (6). Other countries, such as Lebanon, Sudan, and Yemen, have also experienced increased food insecurity after this global pandemic (7).

The global prevalence of moderate and/or severe food insecurity affected approximately 320 million people in 2020, which amounted to the combined number in the past 5 years. This implies that the prevalence of food insecurity drastically increased in 2020 (8). Furthermore, the inability to purchase healthy food increased in Africa and Latin America between 2017 and 2019. This was due to the loss of jobs and shorter working hours, which implies lower wages, higher food prices, falling income, and an increase in the population (8).

The 2021 State of Food Security and Nutrition in the World indicated how attaining zero hunger in 2030 might seem challenging due to the higher undernourishment prevalence, which has increased from 8.4 to 9.9%. This implies a rise in hunger by 46 million people in Africa, by 57 million people in Asia and 14 million people in the Caribbean and Latin America for 2020 compared to 2019 (8).

**Figure 1:**
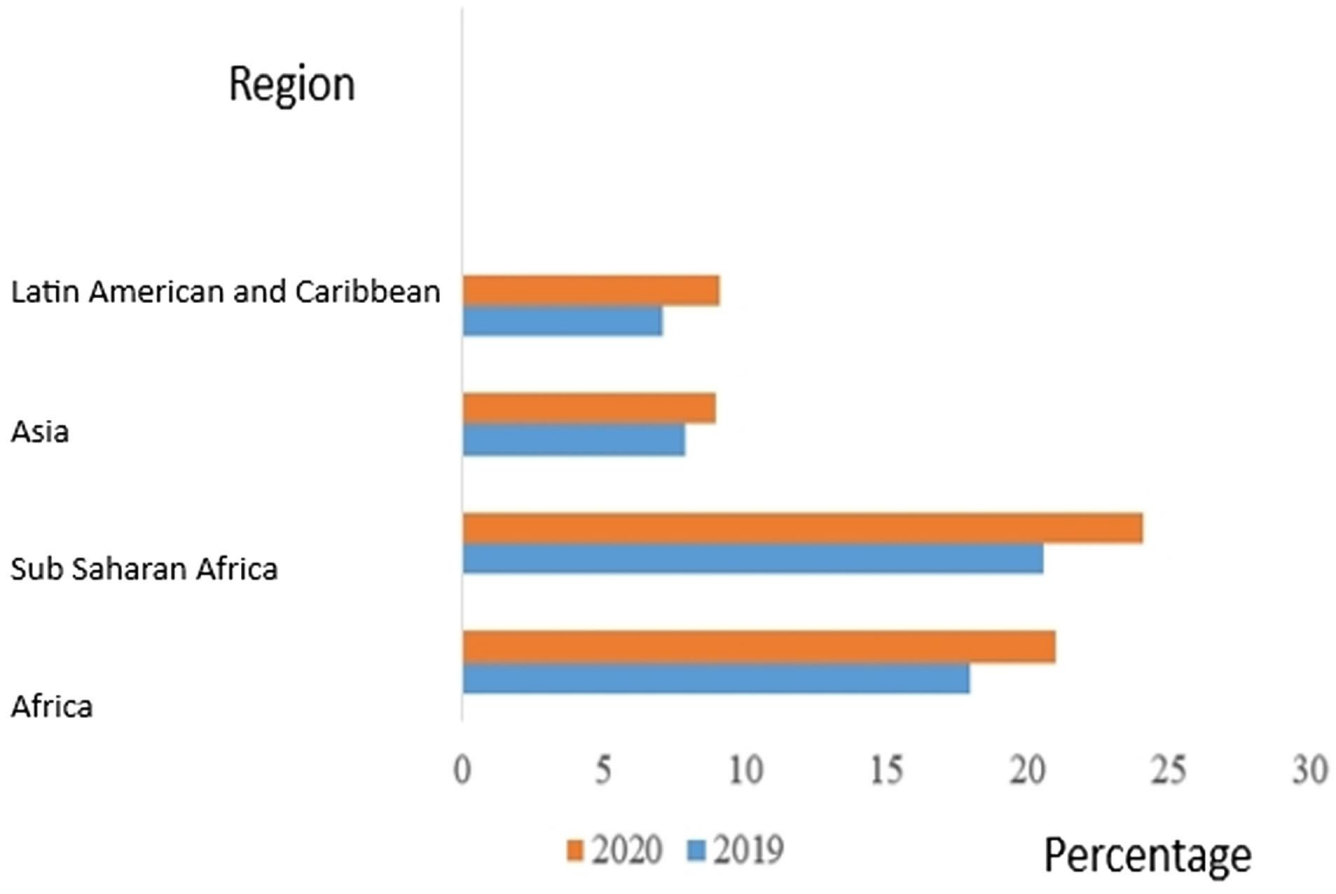
Prevalence of undernourishment by region (Adapted). FAOSTAT (9).

Figure 1 presents the undernourishment prevalence according to regions. The undernourishment prevalence in Africa increased from 18% in 2019 to 21% in 2020, with a rise from 20.6% in 2019 to 24.1% in 2020 in Sub-Saharan Africa (SSA). The increase went from 7.9% in 2019 to 9% in 2020 in Asia, with an increase from 7.1% in 2019 to 9.1% in 2020 in Latin America and the Caribbean (9).

The Food Insecurity Experience Scale (FIES) is the tool used to monitor the rate of moderate to severe insecurity globally.

**Figure 2:**
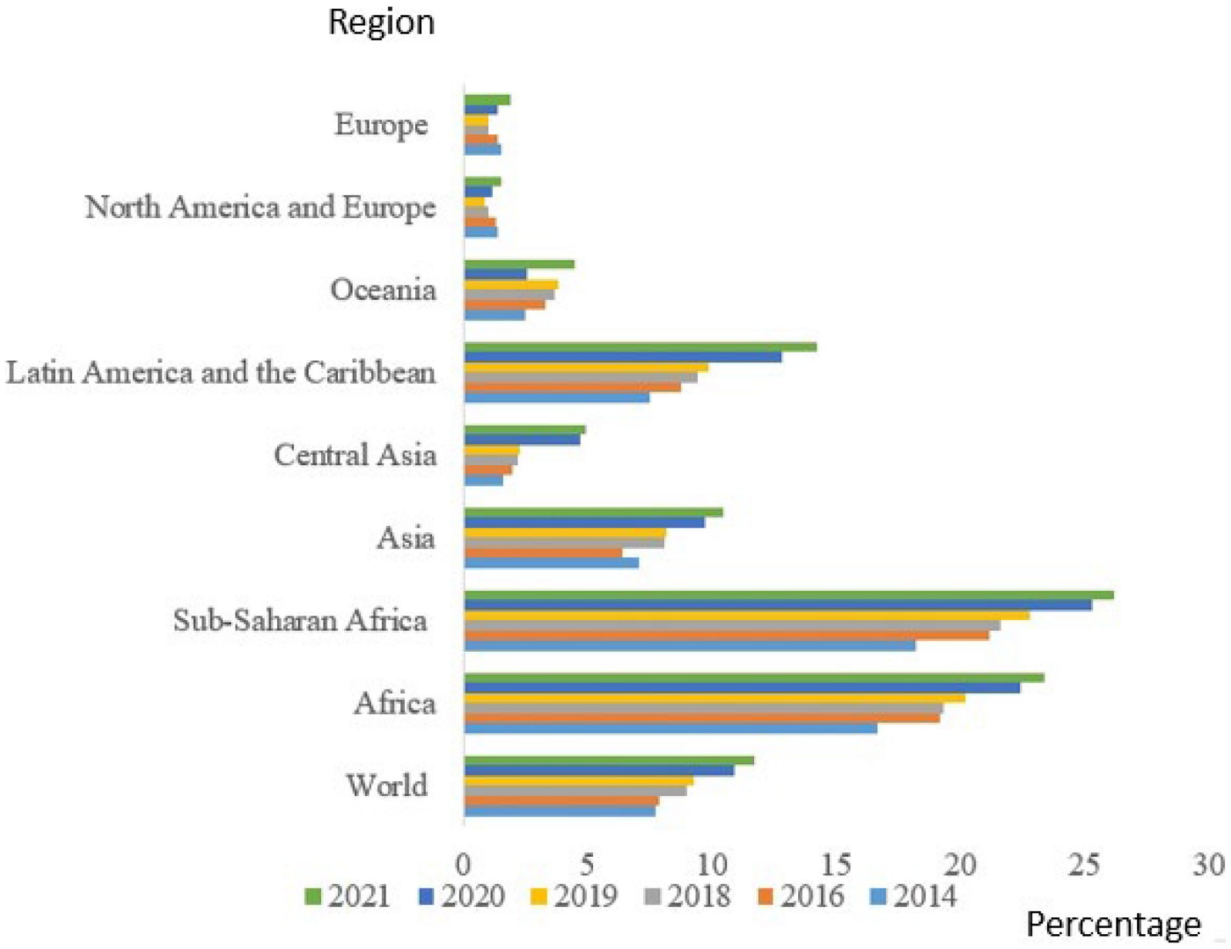
Percentage prevalence of severe food insecurity based on the Food Insecurity Experience Scale, 2014–2021 (Adapted). FAO. 2022 (10).

Figure 2 presents the prevalence of severe food insecurity (percentage). Globally, the prevalence of severe food insecurity increased from 9.3% in 2019 to 11.7% in 2021 – the equivalent of 207 million more people in 2 years. In the same vein, Africa recorded an increase from 20.2 to 23.4%, and in Sub-Saharan Africa, from 22.8 to 26.2%, while in Asia, the level of prevalence increased from 8.2 to 10.5%.

**Figure 3:**
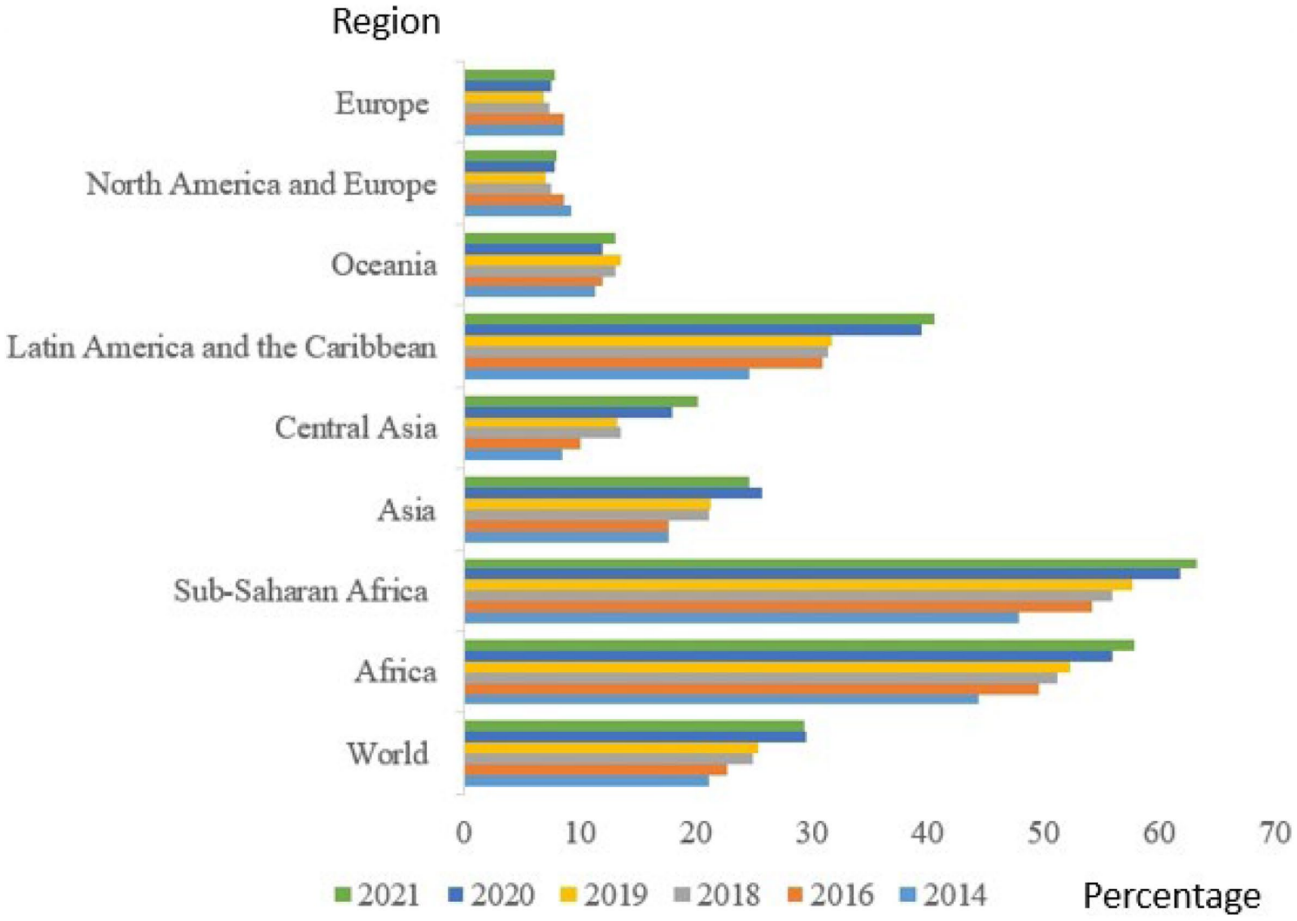
Percentage prevalence of moderate or severe food insecurity based on the Food Insecurity Experience Scale, 2014–2021 (Adapted). FAO. 2022 (10).

Figure 3 presents the prevalence of food insecurity at moderate or severe level, based on the Food Insecurity Experience Scale, 2014–2021. The global moderate to severe food insecurity rate increased from 25.4 to 29.3%. In 2021, it was estimated that about 29.3 percent were moderately or severely food insecure, implying that they did not have access to adequate food. This amount to about 2.3 billion people in the world or nearly 30 percent of the global population. Nearly 924 million people faced food insecurity at severe levels, an increase of 207 million in 2 years. In Africa, there was an increase from 52.4 to 57.9%, and in Sub-Saharan Africa, from 57.7 to 63.2%. In number terms, these resulted in about 928 million people globally being severally food insecure (9). Asides from the pandemic in 2020, which affected food access, food cost, increased poverty, and food insecurity, other factors such as economic challenges, unemployment, non-increment in wages, political instability, terrorism, and climate change had their impact on food and nutrition security. COVID-19 affected not only food availability and access, but also dietary diversity, changes in dietary patterns, and food choices, and increased consumption of unhealthy foods, thus resulting in under-nutrition and non-communicable diseases.

In 2020, nearly 3.1 billion people were unable to afford a healthy diet, which implies an increase of 112 million more people than in 2019. This increase has been mostly driven by Asia, where 78 million more people in 2020 could not afford a healthy diet, followed by Africa with 25 million more people, Latin America and the Caribbean with 8 million more people, and by Northern America and Europe with 1 million more people (10).

The affordability and cost of a healthy diet are useful indicators to denote economic access to nutritious food and a healthy diet and are the core food security principle. Unhealthy/poor diet accounted for roughly 11 million deaths globally per year by 2017, fuelled mainly by rising food prices (4). Moreover, in 2020 early 3.1 billion people were unable to pay for a healthy diet, which increased by 112 million from 2019. These figures indicate the effects of inflation on consumer food prices derived from the COVID-19 pandemic’s economic impacts.

### Population growth, food needs, hunger, and food production

Hunger is one of the world’s major public health concerns. It has been identified that low income & poverty, inadequate governance, war, violent conflict, general lack of freedom, low women’s status, and poorly targeted and delivered health and nutrition programs are the major underlying factors of hunger especially in the low-medium countries. This has greatly hampered the food security of the nations. Although, at the global level, the current food system is not sufficient to address and mitigate these challenges and end hunger. The Global Hunger Index (GHI) is a tool designed to comprehensively measure and track hunger at the global, regional, and country levels, with the ultimate goal of achieving zero hunger in line with the United Nations Sustainable Development Goals. GHI used the three markers: prevalence of undernourishment, child wasting and stunting and child mortality to measure hunger.

Global food insecurity affects 870 million people worldwide, with two-thirds found in Sub-Saharan Africa, India, and China (3). The drivers of global food insecurity are food prices, rising demand and supply for foods due to population growth, climate change, and economic fluctuation leading to inflation and conflicts have been reported to greatly affect low-income countries right from the household level to the national level thereby preventing the world from attaining zero hunger. The severe food insecurity prevalence increased from 9.3% in 2019 to 11.7% in 2021, which is the equivalent of 207 million more people in 2 years. According to the FAO (3), more than 828 million people were affected by hunger worldwide in 2021, which is 46.3 million more people than in 2020 and a rise of 150 million than in 2019. In Africa, 278 million people were affected (20.2% of the population), with 425 million in Asia (9.1% of the population) and 56.5 million in Latin America and the Caribbean (8.6% of the population). Most of the undernourished people on our planet live in Asia, but Africa has the highest malnutrition prevalence (8).

Apart from hunger, millions of Africans present widespread micronutrient deficiencies, where being overweight and obese poses public health concerns in lots of countries.

In 2020, globally an estimated 22% of children aged under 5 were stunted, 6.7% were wasted and 5.7% were overweight. Children from poorer households and rural settings, where their mothers have not received a formal education, were more vulnerable to wasting and stunting. Children from wealthier households and urban areas were at higher risk of being overweight (4).

According to the FAO, a growing undernourishment prevalence has been observed in Africa and at least 256 million people go hungry in Africa (3). It is also worth noting that food production in African continues grows more slowly than population growth. Food production per capita has increased in every other world region except Africa since the 1970s.

The global population is expected to reach 8.5 billion in 2030, 9.7 billion in 2050, and 10.9 billion in 2100, depending upon a range of plausible future trends in fertility, mortality, and international migration United Nations (11). Population growth can also influence food availability and accessibility. This population growth will automatically lead to increased global food demand and consequently increased food production. The population growth rate remains at around 3% annually in SSA, which possibly doubles the number of people in one single generation. FAO estimates that population and economic growth will result in a global increase in food demand 2050 (3). Gbashi et al. (12) confirmed that the population of Nigeria might surpass that of US, with the population of Africa rising by 1.3 billion people with about 41% under 15 years and more than half of the world’s undernourished people are found in Asia (about 418million), Africa about 282 million and 60 million in Latin American and the Caribbean (13). Increased advocacy and education on families’ health needs concerning increased access to family planning are in place to combat increasing population growth in developing countries, but need to be intensified. Increasing financial and political commitments to family planning programs nationally would help to slow down rapid population growth, which would improve families’ healthy living and, consequently, food security.

### Global food security index (GFSI)

This indicates Food security across four pillars namely affordability, availability, quality and safety, sustainability and adaptation. This index is based on a model built from 68 qualitative and quantitative drivers of food security.

In 2022 all regions performed above the global average of 62.2% with North America leading the index with 78.6%, Europe-74.8%, Asia Pacific-63.4%, Latin America-63.4%, Middle East and North Africa-63.0 and 47.0% for sub-Saharan Africa (14).

Affordability, social and political barriers to access have dampened the available the availability of food. Also, GFSI has shown rising risks from armed conflicts and political instability and the Covid-19 pandemic. Some top-ranking countries that are best performers with respect to overall food security environment with respect to 2022 score include Finland-83.7%, Ireland-81.7%, Norway-80.5%, France-80.2%, Netherlands-80.1%, Japan-79.5%, Canada-79.1%, Sweden-79.1%, United Kingdom-78.8%, Portugal-78.7% and the weakest performers include Syria-36.3%, Haiti-38.5%, Yemen-40.1%, Sierra Leone-40.5%, Madagascar-40.6%, Burundi-40.6%, Nigeria-42%, Venezuela-42.6%, Sudan-42.8% and Democratic Republic of Congo-43% (15).

### Food production

The current global production statistics of primary crops based on commodity groups experienced an exponential rise of 52% between 2000 and 2020, which accounted for 9.3 billion tonnes in 2020, and represents 2.9 billion tonnes more than in 2000. In 2020, sugarcane, maize, wheat, and rice made up around 50% of global crop production as the staple crops. In addition, global fruit production rose to 55% between 2000 and 2020, whereas vegetables increased by 65% over the same year. This was followed by sugar crops (23%) and vegetables and oil crops (12% each).

Cereals were the main group of crops produced in 2020, which accounted for about one-third of the total production. Table 1 shows the global cereals yields in tonnes per hectare between 2010 and 2021, including wheat, rice, maize, barley, oats, rye, millet, sorghum, buckwheat, and mixed grains. The table shows that Africa would still experience an upward trend of prevalence of food insecurity because of its lower yield level compared to other countries. The cereal yields observed in South America dropped between 2020 and 2021, while Europe, Asia, and Africa experienced consistently increased in their yields from 2018 to 2021. Agricultural productivity particularly across Sub-Saharan Africa needs to be improved so as to reduce hunger, poverty, and the destruction of biodiversity (17).

**Table 1:** Global cereals yield in tonnes per hectare between 2010 to 2021.

Year	Crop yield (tonnes per hectare)						
	2010	2012	2014	2016	2018	2020	2021
World	3.55	3.61	3.89	4.01	4.03	4.12	4.15
South America	4.15	4.26	4.50	4.47	4.62	5.07	4.68
Europe	3.71	3.90	4.36	4.21	4.24	4.35	4.54
Asia	3.64	3.63	3.95	4.10	4.22	4.28	4.31
Africa	1.59	1.57	1.59	1.53	1.67	1.71	1.75

Adapted from Our World in Data (2022) based on World Bank; Food and Agriculture and Organization of the United Nations (16).

Fruits, roots, and tubers each accounted for 9–10 percent of the total production. The increase in food production over this period is mostly attributed to some enabling factors, which included improved farming practices, increased use of irrigation, pesticides, and fertilizers as well as improved and high-yield crops (3). Most primary crops can be consumed unprocessed, but two crop groups, in particular, require processing for the production of commodities that are used as both food and fuel. They, therefore, have an impact on nutrition and health as well as on energy and the environment.

Food loss has been reported to greatly impact the economy, environment and livelihoods of Sub-Saharan-African (18). Most food loss occurs on the field and post-harvest especially in low-income countries and in Sub-Saharan Africa. Cereal grains, such as wheat, rice, and maize are the most popular food crops in the world are the basis of staple foods in most developing countries. Kumar and Kalita (18) substantiated that minimizing cereal losses in the supply chain could help in regards to strengthen food security, sustainably combat hunger, reduce the agricultural land needed for production, and rural development, and improve the farmers’ livelihood. Post-harvest loss accounts for the direct physical losses and quality losses that reduce the economic value of the crop, or they may make it unsuitable for human consumption. These losses can be up to 80% of the total production in severe cases (19). These losses have been estimated to range between 20 and 40% in African countries, which is highly significant considering the low agricultural productivity in several regions of Africa. About 1/3 of the food produced annually for human consumption is lost or wasted. Food losses and waste were approximately $680 billion in industrialized countries and around $310 billion in developing countries (19).

**Figure 4:**
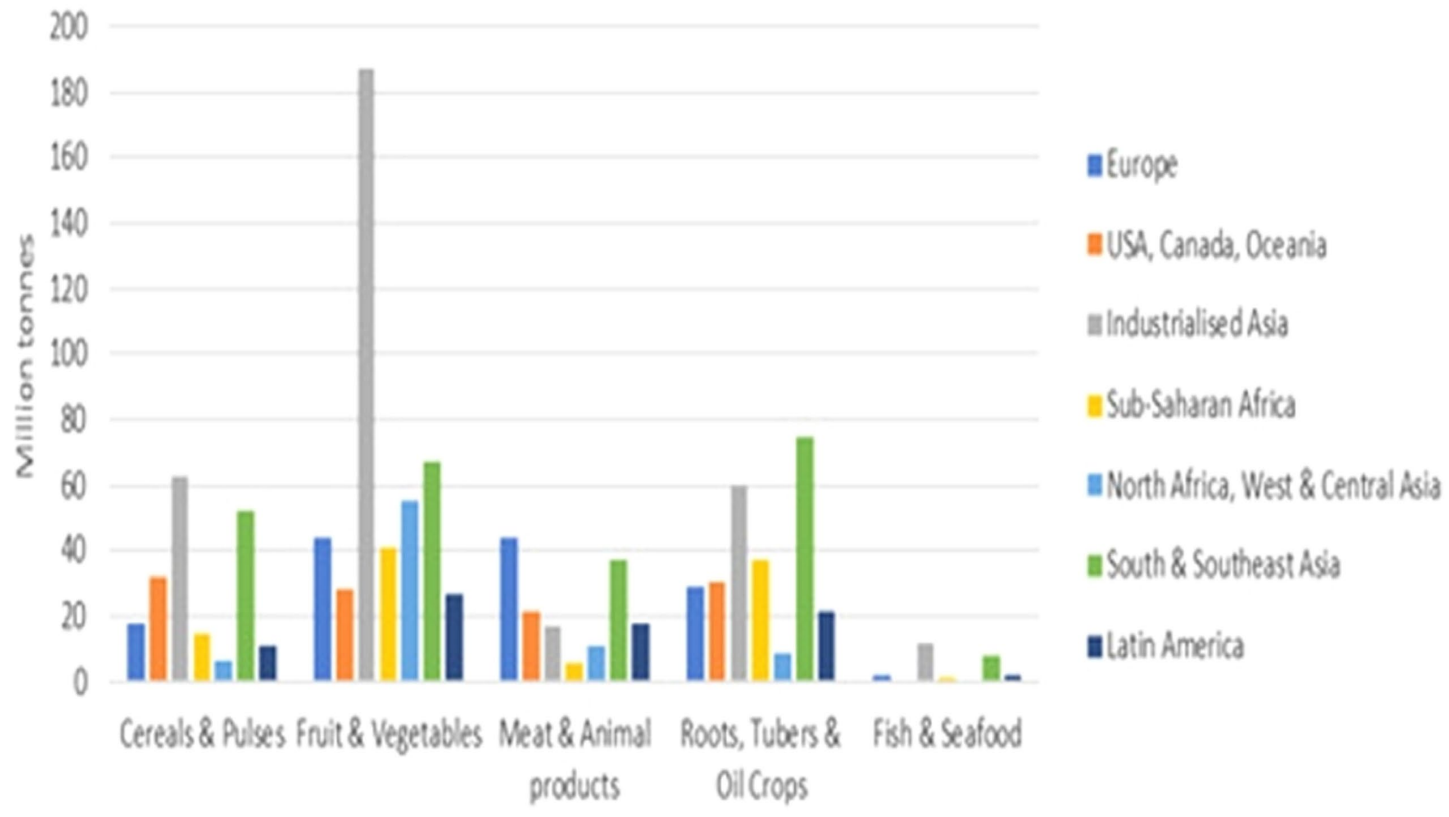
Food losses by commodity and region (million tonnes). WWF Technical report (20).

Figure 4 shows food losses per commodity and region. Observations for fruits and vegetables losses are the most prevalent in Central and industrialized Asia, whereas about 80 million tons of roots, tubers and oil crop loss were for South and Southeast Asia. The losses for fruit and vegetables, roots and tubers, and oil crops, are estimated to be 40%, with about 38% and about 25% in Sub-Saharan Africa. According to Sawicka (21), the annual global food losses and wastes were estimated to be approximately 30% for cereals; 40–50% for root crops and fruit and vegetables; 20% for oilseeds, meat, and dairy products; and 35% for fish. Report also confirmed that consumers in developed countries lose almost as much food as over 220 million tons as the total net food production in sub-Saharan Africa which is around 230 million tons, thus making the total amount of food lost or wasted annually equivalent to more than half of the world’s annual crop production which was about 2.3 billion tons per year. Reducing food loss and waste is an important target of the Sustainable Development Goals (SDGs) as well as a means to achieve other SDG targets that are particularly related to food security, nutrition, and environmental sustainability.

### Value addition: a critical tool for food/nutrition security

Consumers now focus more on convenience, variety, health, wellness and social awareness, and place more value on their time. Coltrain et al. (22) define value addition as” to economically add value to an agricultural product, such as wheat, by processing it in products, such as flour, which are desired by customers, such as bread bakers. Value addition can equally be regarded as a worthwhile investment because it generates a higher return, which allows the penetration of a new potentially high-value market, extends the production season, creates a brand identity, or develops brand loyalty for consumers/customers.”

In addition, it can be defined as the additional features of economic value that an industry adds to its products and services before offering them to consumers.

First, consumer behavior and demand for processed food have increasingly changed in recent years because of prevailing societal and environmental challenges. Second, the increasing population of developing countries, including Nigeria, and poor households with no access to nutrient-dense foods, are important factors to consider in order to eradicate hunger and malnutrition, and objectives that move toward a sustainable agenda for enhanced global food and nutrition security. Value addition is an important tool in promoting diet diversification and could, therefore, be achieved by low-cost food processing and proper preservation/storage techniques, which would greatly reduce global food loss. Appropriate processing technologies must be developed to minimize postharvest losses to develop sustainable value-addition systems for improved nutritional health security to, in turn, minimize hunger and to create more job opportunities. In addition, appropriate infrastructural facilities, such as good transportation systems for easy access to raw materials and finished food products to consumers, would be an impacting intervention as regards improved value addition systems.

However, the global value addition brought about by agriculture, forestry and fishing grew by 68% in real terms from 2000 to 2018. In 2018, this came to USD 3.4 trillion, and represents an increase in USD 1.4 trillion versus 2000. Value addition more than doubled for the same period in Africa, with an increase from USD 170 billion to USD 397 billion. The report shows Asia as the main contributor to global agriculture because of its size, while the value addition for forestry and fishing was 63% of the world’s total in 2018. This continent obtained an increase of 77%, which was a rise from USD 1.2 trillion in 2000 to USD 2.2 trillion in 2018. The increase for the Americas was 47%, and that of Oceania was 21% during the 2000–2018 period. Europe’s agricultural value addition rose by 18% from USD 278 billion in 2000 to USD 329 billion in 2018. In 2018, the countries with the biggest agriculture, forestry and fishing sector in value addition terms were India, China and the United States (9).

### Role of food processing industries in food/nutrition security

Food processing entails transforming raw ingredients. The preservation techniques of the resulting products make them palatable and extend food’s shelf life for consumption (23). Processing increases the variety of food products made available to consumers, and makes them more convenient, which meets their immediate demands. Moreover, food processing creates excellent income and job opportunities for people, which in turn would enhance the purchasing power of consumers and consequently improved food affordability and accessibility (24). Food processing industries are engaged in both the primary and secondary processing of agricultural products for subsequent human use or consumption. Processing can have significant effects on effective food supply, and can be considered not only an integral part of today’s diet, but also a significant contributor to food/nutrition security (25). Food/nutrition security relies on the apt supply of affordable, safe and nutritious fresh and processed food to everyone. Increasing food production by means of food-processing techniques would help with the challenge of supplying a healthy diet to 9 billion people in 2050. Food processing promotes and enhances preservation, nutrient quality, safety and the shelf life of foods.

Major conventional and emerging process technologies can be applied to develop products for food processing and in the supply chain. Emerging technologies, including high-pressure processing (HPP), pulsed electric field (PEF), cool plasma, ultraviolet irradiation and ultrasound, have been explored in the food industry as treatments that prolong the shelf life of foods (25, 26). Applying emerging and non-thermal techniques can potentially cut energy requirements for food processing and may contribute to enhance the food industry’s energy efficiency (27).

The need to improve resource efficiency in food processing industries cannot be overemphasized because most food processing industry operations are energy-intensive, and they do not optimize employing edible agricultural food sources. Many of today’s agricultural practices rely on cheap energy, and need plenty of water and land, and are a leading source of greenhouse gas (GHG) emissions. Food processing industries can, therefore, integrate the complete use of fewer resources along the whole food value and supply chain from farms to consumers’ table (28).

The application of technological education via science and technology in food production, such as mechanized farming, and biotechnology that is genetically modified food, and training agricultural entrepreneurs in basic food production techniques, could also improve food availability and utilization, and overall agri-food systems. In addition, the application of research and development innovations by collaborations with academia, research institutes and food processing industries could help to improve sustainable food/nutrition security.

### Genetically modified food in food security/nutrition

The prevalence of hunger situations in nations around the world, and the ravaging impact of COVID-19 on their economy and population outbursts, have involved considering effective food systems to promote food/nutrition security, and are extremely important. Modern approaches have been developed in the biotechnology field to enhance food/nutrition security. Genetically modified crops resort to many modern approaches, which are applied to improve existing agrifood systems. Genetically modified (GM) foods employ biotechnological processes to alter the inherent features of living organisms by replacing the natural gene sequence.

The FAO defines a GMO (genetically modified organism) as an “organism where one gene or more, called transgenes, have been introduced into its genetic material from another organism using recombinant-deoxyribonucleic acid (r-DNA) technology” (29). GM foods are those deriving from organisms whose genetic material (DNA) has been modified in such a way that it does not naturally occur; e.g. by introducing a gene from a different organism. This is known as genetic engineering technology (30). Such food is becoming increasingly common in many developed and in a few developing countries. Rice, maize, potatoes, wheat and soybean lie at the top of the list of products from GM species. GM foods provide a range of opportunities: increased food production locally and globally, climate adaptability and tolerance, which would, consequently, improve food availability at all levels and create better food options. According to the World Bank (30), GM crops could also improve food nutritional quality and safety, which would positively influence economic access to food. Other beneficial outcomes of GMO foods include longer shelf life, preventing allergies and diseases, malnutrition and food security. Gbashi et al. (12) provided details of GMOs’ success to combat food production shortages and losses, as in crop failure due to climate change, pathogen/disease infestation, poor crop yields, late harvests and delayed maturity. These opportunities were achievable using several genetic modifications of existing crops (i.e., GM insect-resistant maize with healthier cobs), which are infested by mycotoxins much less and offer significant health benefits. In addition, cowpeas, which are pod-borer resistant cowpeas called SAMPEA 20-T, and pest-resistant Bt cotton and golden rice, have been produced by genetic engineering to biosynthesize beta-carotene, a precursor of vitamin A, in edible golden rice parts (31, 32). They are all genetic engineering technology success stories for sustainable agriculture systems.

### Unaddressed identified gaps/challenges in the global food/nutrition security system

Malnutrition remains a challenge despite several measures being put in place to tackle it at all levels. In 2020, it was estimated that about 149.2 million children under the age of 5 were stunted, 45.4 million were wasted and 38.9 million were overweight. These statistics were for children from Africa and Asia, and these numbers are expected to rise due to the COVID-19 pandemic (8). Another challenge is to meet the global nutrition targets for 2025 and 2030 as regards wasting, overweight children, adulthood obesity and anemia in women and children. There is still a long way to go to meet these targets by 2030, whereas improvements in child stunting, low birth weight and exclusive breastfeeding still seem inadequate (8).

The burden of foodborne diseases on public health and economies has often been underestimated, especially in low-medium income economies. Children under the age of 5 are the most vulnerable, with over 40% suffering foodborne illness, which results in 125,000 deaths every year (4). The 2019 World Bank also reported a huge total productivity loss in the economy of low-income and middle-income countries because of foodborne diseases, and was estimated at 95.2 US$ billion per year, with the annual cost of treating illnesses estimated at 15 US$ billion. Safe food supplies support sustainable national economies, trade and tourism and, consequently, contribute to food/nutrition security.

Food systems have multifaceted impacts on food/nutrition security as regards ensuring inclusiveness and food sustainability. Food system drivers are essential in their transformation, and will improve food availability and affordability (11). Individuals, which is the private sector, and the state play a fundamental role as regards the food sector, and they must work together to reduce food insecurity worldwide. Individuals have guaranteed their right to access food and nutritional need, which contributes to food production. The private sector improves income support, provides food production and amends laws for society. The state implements public policies, provides education and supports the right to food. Food security needs national and international strategies concerning investments to provide employment, to minimize natural and human-made disasters, to adapt to sustainability, to set up economic development policies and to improve access to suitable food.

### Agricultural transformation for sustainable food security

In order to lift millions out of poverty, it is required that smallholder agriculture must be highly productive and profitable, especially in the developing countries of the world. In order to achieve this goal, a stable framework of macroeconomic political stability are essential, effective technology transfers system (33), good access to lucrative markets, ownership system which includes a system of enforcing rights that rewards individual initiative and toil, and employment creating non-agricultural sectors (33). Additionally, national policy makers are potentially benefitting from adequate and convincing information on successful and relevant experiences that have led to intended transformation. In Nigeria in the ongoing ATA, for farmers to utilize and apply innovation generated by the knowledge/technology generating subsystem there must be an efficient technology transfer sub system (34).

In Asia-Pacific, a large number of conducted studies have been linked to specific public policies and interventions to successful agricultural transformation. National policy made are likely to benefit significantly from adequate and convincing information on successful and relevant experiences in successful transformation. The key public sectors interventions relevant to sustainable transformation generally include policies, legislation, and institutional interventions (35). The vast majority of Latin American and Caribbean countries are not importers of cereals. In this region, 26 countries are highly reliant on Wheat imports while 13 countries are also highly reliant on maize imports. Regional imports of wheat, maize, vegetable oil and other foodstuffs are being affected by the war in Ukraine through higher global prices. Caribbean sub- region importer of most food commodities such as maize, wheat, dairy products, fruits, vegetables, meat, vegetable oil etc. (36). There is also insufficient fertilizer production leading to high cost of fertilizer in this region as a fallout of the war between Ukraine and Russia (2018–2022) as they depend highly on fertilizer imports. Russia and Ukraine contribute about 2% of the global Gross Domestic Product (GDP), they are both food basket, producing and exporting essential agricultural commodities, minerals, fertilizers and energy (36). Before the conflict between Russia and Ukraine, Russia was the world’s largest supplier of fertilizers (such as Nitrogen, Potassium, and Phosphorus). This has led to increase in prices of food which might adversely affect global food security especially in vulnerable countries including the European food system which has also been adversely affected by the COVID 19 pandemic (37). There is therefore a need for Latin America and Caribbean countries to promote agriculture through favorable national policies, produce fertilizers at affordable price to farmers and also support food value chain activities for food and nutrition security as a means of reducing imports, improved job creation and enhancing Food and Nutrition security in this region.

### Role of functional food and nutraceuticals in food/nutrition security

Functional foods play important roles in food/nutrition security. A functional food is a food with specific beneficial effects on one or more target functions in the body that go beyond basic nutritional functions and result in improved health status and well-being as well as reduction in the risk of disease. Given this, functional foods have raised consumers’ interest toward healthy diets thereby occupying a major place in the global food market. Some functional foods are plant-based and contain bioactive components (i.e., flavonoids, catechins, carotenoids, lutein, lycopene, phenolics, quercetin, anthocyanidins) with health potentials (38). Some natural food products such as millet (39), mushroom (38), microalgae (40), flaxseed, oat, oranges, soybeans, onions and cherries (38) have been found to contain bioactive components with potential health benefits. Fruits and vegetables are another example of functional foods which are rich sources of essential bioactive compounds including carotenoids and polyphenols which have beneficial health properties.

Millet is rich in iron, calcium, fiber (12%), protein (7–11%) and bioactive compounds, which make it a sustainable food security crop (39, 41). It grows under harsh-growing conditions and can be included in infant meals to provide them with iron, calcium and protein, and for the nutrition of women of reproductive age to prevent anemia.

Mushrooms are an excellent source of proteins (13–39% dry weight), fiber, minerals and vitamins, and are low in sodium, lipids (1.18–8.39% dry weight), and calories (42, 43). They contain phenolic compounds: i.e. tannins, lignans, flavonoids, and phenolic acids. Mushrooms are known for their medicinal and nutritional benefits. They have been reported to possess anti-tumor, antioxidant, anti-diabetic, anti-obesity and anti-viral potentials (44, 45). They also possess prebiotic properties, which improve gut health (45).

Microalgae are rich sources of protein, carbohydrates, vitamins A, B1, B2, B6, B9 and E, and minerals like iron, potassium, calcium and magnesium. The most consumed microalgae are spirulina and chlorella. They are used in tablets, and in liquid and powder forms in food and drink. They possess properties that can treat the heart, eyes and cancer diseases. They have various food industry uses as food additives and pharmaceuticals, and also for skin care (44, 46).

These functional foods have favorable growing conditions. Better exploring the production of these food crops will increase food production, sustainability, access and healthy diet.

These functional foods have been found to possess various health benefits, such as the cardio-protective effect, and possess anti-oxidant, anti-inflammatory, anti-estrogenic, anti-viral, anti-obesity, anti-diabetic and anti-tumor potentials (45–47). Consumption of functional foods with a healthy lifestyle can make a positive contribution to health and wellbeing which aligns with the United Nations Sustainable Development Goal Number 3.

### Role of underutilized food crops in food/nutrition security

The benefits of underutilized food crops cannot be overemphasized for food security. Most of these food crops possess unique features, which allow them to tolerate harsh weather conditions, drought and soil types with high yields. Enset (*Ensete ventricosum, Musaceae*) is similar to bananas, which are cultivated and consumed in Ethiopia. It is high-yielding and can be harvested at any time. Its starch is usually extracted, fermented and consumed. It is an underutilized crop that can help to solve food/nutrition security (48). Another crop identified in Ethiopia with the potential to solve food/nutrition security is *teff*. Teff (*Eragrostis tef*) is nutritious, and is high in dietary fiber (9.8%), protein (9.37%) and minerals, such as phosphorus (429 mg/100 g), magnesium (184 mg/100 g), calcium (180 mg/100 g), potassium (427 mg/100 g), iron (7.63 mg/100 g), sodium (12 mg/100 g) and zinc (3.63 mg/100 g), as well as polyphenols, (37–39) and contains dietary fiber with proteins minerals and polyphenols, and is gluten-free (49, 50). It has been utilized for malting and brewing, and also for non-gluten food and beverage production (51), and for making bread, cookies, cakes, fermented cereals, and other confectionary products (52).

Amaranth (*Amaranthus* spp.) is an underutilized grain classified as a pseudocereal. It can be a cream, which is a pink or brown, and can grow under harsh weather conditions. It is an indigenous high-yielding vegetable rich in sulfur-containing amino-acids, lysine and minerals. It can be eaten as a leafy vegetable or grain depending on the species (53). Studies show that it possesses a health-promoting potential against cancer. It is anti-hypertensive, anti-diuretic and anti-inflammatory, and lowers cholesterol levels (53, 54). It is rich in folate, calcium, carotene, protein and vitamin C (55). Amaranth has about 13.56% protein, 7.2% lipids, 2.88% ash, 6.7% fiber, 65.25% carbohydrates, 7.61 mg/100 g iron, 159 mg/100 g calcium, 508 mg/100 g potassium, and 248 mg/100 g magnesium, which is higher than cereals like maize, sorghum, rice, and wheat (55, 56). It can be consumed dried and fresh as vegetables, and can be roasted or milled, and used for bread making, salads, cookies, porridge, *kunu* (57), and also in infant complementary food production (58). Amaranth entails various food, industrial, pharmaceutical and cosmetic uses (58). It is grown mostly in Mexico, India, China, Russia and Kenya. Some of the challenges that affect exploring amaranth include its cultivation, processing it into value-added products and its distribution.

Quinoa (*Chenopodium quinoa Willd*.), which is referred to as *golden grain*, is mostly cultivated in South America. It is a drought-, cold-and salt-resistant crop that can survive under extreme and harsh climate conditions. It is high in protein (11–19 g/100 g) with 18% total dietary fiber (59). It also has an excellent amino acid profile compared to other grains. Its fat content is 2–9.5%, which is higher than other grains, except soybeans. It contains high mineral contents, such as calcium (148.7 mg/100 g), zinc (4.4 mg/100 g), potassium (926.7 mg/100 g), iron (13.2 mg/100 g) and magnesium (249.6 mg/100 g). It is rich in riboflavin (0.2–0.3 mg/100 g), thiamine (0.2–0.4 mg/100 g), niacin (0.5–0.7 mg/100 g) and folic acid (0.08 mg/100 g) (59).

Other food security crops with such a potential are bambara groundnut (60), African breadfruit (61), mungbean (62) and pumpkins (63).

Bambara groundnut (*Vigna subterranea L. Verdc*) is an underutilized African legume. It contains 23.6 g/100 g protein and 5.5 g/100 g dietary fiber (64). It is commonly consumed in Eastern Nigeria as *okpa*, which is a meal made of its flour and boiled with other ingredients. It can made into milk, condiments, infant weaning food, bread and cookies (65, 66).

African breadfruit (*Treculia africana* Decne) is common in South Eastern Nigeria. It is eaten roasted, dried into flour, as a soup thickener, as beverage and alcoholic drinks, and as porridge or complementary food. Its leaves are rich in phytochemicals and are employed for various medicinal purposes (i.e., anti-bacterial and anti-inflammatory). It is also used as fodder and livestock feed (61).

Moringa (*Moringa oleifera*) is a potential underutilized functional food. It is rich in proteins, vitamins and bioactive compounds. It has antioxidant, anti-cancer, anti-diabetic and anti-inflammatory properties (67, 68). It is also a food security crop because its seeds, leaves, flowers and pods are good nutritional food (69). Fresh moringa leaves contain about 6% protein, with 17% for processed leaves. Fresh seeds have 13% protein, whereas processed seeds contain about 38% protein. Drying and fermentation processes reduce the anti-nutritional content, such as phytate, oxalte, tannin and saponin, in moringa leaves, and seeds (69).

Mungbeans (*Vigna radiata* var. *radiate*) are rich in iron (9-11 mg/100 g), folate (31.36 ppm) and protein (20.97–31.32%), which makes them suitable to be used with other cereals as complementary food and in vegan diet (70–72). As their growing period is short, they can be grown many times in a year. It is also a nitrogen-fixing crop (73).

Pumpkins (*Cucurbita* spp.) are produced mostly in China and are highly nutritious. Pumpkin peel, flesh and seeds are rich in minerals like calcium (0.62–1.33 g/100 g), iron (8–18 mg/100 g), zinc (1.72–5.17 mg/100 g), potassium (2–4.81 g/100 g), fiber (20–73%), phytochemicals and vitamin A total carotenoid content (94–3,038 μg/g) (74). Pumpkins have various food and pharmaceutical uses. They possess anti-bacterial, anti-cancer, anti-inflammatory, anti-diabetic and anti-hypertensive potentials (75). Pumpkins are consumed as jam, juice and dried foods (75).

These food crops will drive sustainability, reduce malnutrition and improve diet diversity. They will enable crop diversification and reduce over-relying on maize, rice, and wheat, and shift the concentration on some of these highly nutritious alternatives. Furthermore, commercializing their cultivation will generate income and improve economic activities (62, 64).

### Role of food regulatory bodies in food/nutrition security

Food and regulatory bodies play several roles to ensure food/nutrition security. Various laws, guidelines and regulations had been promulgated to ensure the safety and wholesomeness of foods right from the farm to the table. Food regulatory bodies are the agencies established to ensure the quality, safety, and health of food through public education and enforcement of the established laws and regulations. They ensure that standard procedures are followed and complied with in the production, processing, distribution, transportation, and handling of food products. These regulatory bodies/agencies vary from country to country. At the global level, there are food quality control legislation bodies including Codex Alimentarius Commission (CAC), World Trade Organization (WTO) and International Standard Organization (ISO), among others which were established to set internationally agreed guidelines, standards to improve national food quality control and safety management systems. Laws and guidelines should be implemented to improve food safety in food value chains to reduce food loss and waste and to guarantee that all drivers of food systems conform to standards to foster healthy nutrition outcomes. Nutrition education should be emphasized, especially to promote organic home gardening of vegetables by households. Furthermore, food regulatory bodies should advocate and implement the cultivation of healthy seeds for healthy food crop production. They should promote the large-scale indigenous production of underutilized food crops and support their trade and exportation. The additional incorporation of safe ingredients with functional properties deriving from plant sources in food formulations/recipes should be promoted by industries, and ought to be regulated to conform to already established standards or a set of new standards for conformity purposes.

Regulatory bodies must be aligned with sustainability pillars to promote food and nutritional security by contemplating the two major and important food supply factors: natural resources and agriculture. The three most cited dimensions are economic, social and environmental. The use of clean technologies and environment-friendly products, waste treatment and destination, recycling, social responsibility in food production, community growth generation and involvement in social projects are required. This can be achieved by considering the economic aspects of food production and access (76, 77). Some authors include others, which are associated with sustainability applied to culture nutrition and health, and they go beyond these dimensions. The authors realize that sustainable nutrition can affect human health on the health dimension, and they indicate respect for individuals’ cultural origins and eating habits on the cultural dimension (77, 78).

### Recommendations/way forward

Food/nutrition security is multifaceted. However, the challenges that emerge can be overcome by promoting the diversification of these highly nutritious food crops with potential health benefits. Improving value addition and the utilization of these crops will lead to people being aware of them and will generate income for good economy.

## Conclusion

By way of conclusion, the global food security report reflects the true state of the world, and it is important that every stakeholder involved in food and nutrition policies, regulations and the agric-food chain all make efforts to ensure zero hunger and to guarantee healthy well-being. Critical food safety programs for food handling procedures that can prevent foodborne diseases are essential to ensure an active healthy lifestyle. Furthermore, the identified underutilized crops and functional foods should be explored for their food and nutritional benefits to foster diet diversification and to promote global consumption of nutritious and healthy food. Public knowledge of and awareness about GMOs will play a major role in consumer education and acceptability, in addition to government interventions, in terms of regulations and safety assessments, which will strengthen options about creating food security, particularly in developing countries.

## Author contributions

All authors listed have made a substantial, direct, and intellectual contribution to the work and approved it for publication.

## Conflict of interest

The authors declare that the research was conducted in the absence of any commercial or financial relationships that could be construed as a potential conflict of interest.

## Publisher’s note

All claims expressed in this article are solely those of the authors and do not necessarily represent those of their affiliated organizations, or those of the publisher, the editors and the reviewers. Any product that may be evaluated in this article, or claim that may be made by its manufacturer, is not guaranteed or endorsed by the publisher.
